# Unveiling the photoluminescence dynamics of gold nanoclusters with fluorescence correlation spectroscopy†

**DOI:** 10.1039/d3na00869j

**Published:** 2023-12-04

**Authors:** Malavika Kayyil Veedu, Julia Osmólska, Agata Hajda, Joanna Olesiak-Bańska, Jérôme Wenger

**Affiliations:** a Aix Marseille Univ, CNRS, Centrale Med, Institut Fresnel, AMUTech 13013 Marseille France jerome.wenger@fresnel.fr; b Institute of Advanced Materials, Wroclaw University of Science and Technology Wrocław Poland

## Abstract

Gold nanoclusters (AuNCs) have captured significant interest for their photoluminescent properties; however, their rapid photodynamics remain elusive while probed by ensemble-averaging spectroscopy techniques. To address this challenge, we use fluorescence correlation spectroscopy (FCS) to uncover the photoluminescence dynamics of colloidal Au_18_(SG)_14_ nanoclusters. Our FCS analysis reveals the photoluminescence (PL) brightness per nanocluster, elucidating the impact of photoexcitation saturation and ligand interactions. Unlike DNA-encapsulated silver nanoclusters, their gold counterparts notably exhibit minimal blinking, with moderate amplitudes and 200 μs characteristic times. Our data also clearly reveal the occurrence of photon antibunching in the PL emission, showcasing the quantum nature of the PL process, with each AuNC acting as an individual quantum source. Using zero-mode waveguide nanoapertures, we achieve a 16-fold enhancement of the PL brightness of individual AuNCs. This constitutes an important enabling proof-of-concept for tailoring emission properties through nanophotonics. Overall, our study bridges the gap between ensemble-averaged techniques and single-molecule spectroscopy, offering new insights into AuNC photodynamics for biosensing and imaging applications.

## Abstract 2

Atomically-precise gold nanoclusters, known for their unique photoluminescence, are rarely explored at the single nanocluster level. Our study using fluorescence correlation spectroscopy reveals key insights into their photoluminescence dynamics.

## Graphical abstract

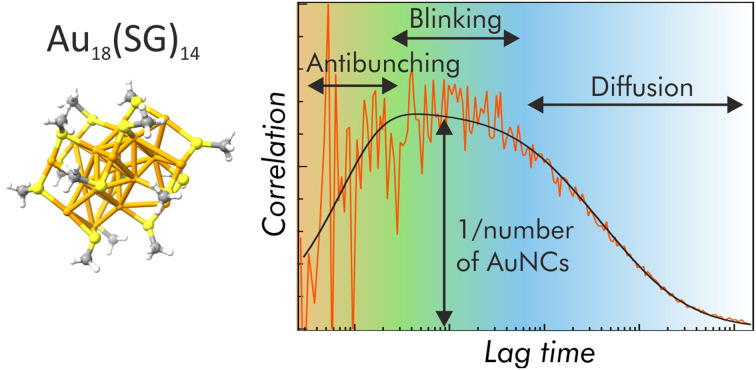

## Introduction

Gold nanoclusters (AuNCs) represent a specialized class of atomically precise metal nanoparticles (NPs) with diameters typically below 2 nm. This small size attributes the nanoclusters with unique electronic and optical characteristics that diverge from larger nanoparticles, primarily due to their high surface-to-volume ratios, electron sharing mechanisms, and quantum confinement effects.^1,2^ Existing at the interface between individual atoms and larger metal structures, gold nanoclusters offer crucial insights into progressive changes in the structure and properties that occur when scaling from atomic to nanoparticulate forms.^3^ A noteworthy feature of AuNCs is the concept of a ‘superatom’, which refers to specific combinations of gold atoms, surface ligands, and charges that result in particularly stable nanocluster structures with closed electron structures.^4^ Nanoclusters manifest molecular-like behavior, including discrete electronic structures and notably distinct optical properties, featuring luminescence ranging from visible to near infrared wavelengths, which originates from the S–Au–S–Au–S semi-ring states and Au core state transitions.^5–7^ As a result, AuNCs have gained significant attention for a multitude of applications, ranging from catalysis,^8,9^ to optical devices,^10–12^ biosensing,^13^ and bioimaging.^14,15^

While gold nanoclusters are attracting large interest, their photoluminescence (PL) properties remain primarily scrutinized through bulk ensemble-averaged spectroscopy. This approach, unfortunately, fails to unveil rapid photodynamic processes occurring below the millisecond time scale, leaving critical questions unanswered. Does the AuNC luminescence exhibit blinking behavior? If so, at what time scale? How does the brightness per nanocluster evolve with the excitation power? How many emitting centers are present on an AuNC? Ensemble measurements are unable to resolve fast photodynamic processes, prompting the need to apply techniques from single-molecule fluorescence spectroscopy in order to find answers.^16,17^ Among different single-molecule techniques,^18^ fluorescence correlation spectroscopy (FCS) is a versatile and powerful tool to investigate all the dynamic phenomena leading to a change in the fluorescence intensity, from photon antibunching to triplet state blinking and translational diffusion.^19,20^

Here, we apply FCS to unveil the photoluminescence dynamic properties of water-soluble Au_18_(SG)_14_ nanoclusters (SG – l-glutathione), with well-defined structures and good PL properties.^21,22^ Our FCS data assess the PL brightness per individual nanocluster, allowing the transition into the saturation regime to be properly reported while increasing the excitation power. Our findings demonstrate that heavy water as solvent and pyridinedicarboxaldehyde (PDA) as a ligand passivating shell significantly promote PL brightness. Contrary to DNA-encapsulated silver nanoclusters,^23^ these gold nanoclusters exhibit moderate blinking, with amplitudes below 0.2 and characteristic times around 200 μs. Furthermore, our observations reveal the signature of photon antibunching in the nanocluster emission, highlighting the quantum nature of the PL process. This insight firmly establishes each AuNC as an individual quantum source featuring a single emitting center. Shifting away from the conventional confocal microscope system, we show that zero-mode waveguide nanoapertures of 110 nm milled in an aluminum film can enhance the PL brightness of individual AuNCs by an impressive 16-fold factor. This proof-of-concept expands our understanding and paves the way for using nanophotonics to tailor AuNC emission properties. Gaining deeper insights into the AuNC photodynamics beyond the capabilities of ensemble-averaging techniques represents a critical stride toward future advancements in deploying AuNCs for biosensing and imaging applications.

## Results and discussion

### Confocal measurements of Au_18_(SG)_14_

**Fig. 1:**
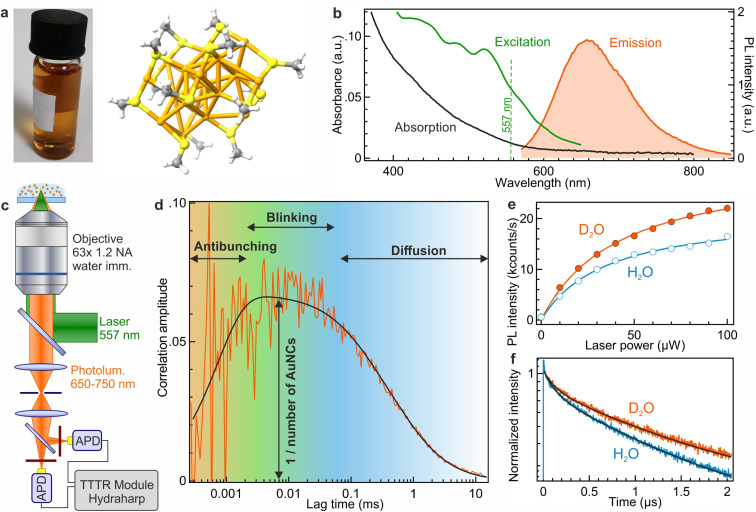
(a) Vial containing Au_18_(SG)_14_ used for the measurements and schematic representation of the AuNC (orange: gold atoms and light yellow: sulfur, instead of glutathione, only simplified functional groups –SCH_3_ are represented). (b) Absorption, excitation, and emission spectra of Au_18_(SG)_14_. We used an excitation laser of 557 nm for further experiments and APDs collecting photons in the range 650–750 nm. (c) Scheme of the FCS setup used for our measurements. (d) FCS correlation curve of Au_18_(SG)_14_ illustrating various photophysical processes occurring at different time scales. (e) Comparison of the photoluminescence (PL) intensity and fluorescence decay trace of Au_18_(SG)_14_ dispersed in H_2_O and D_2_O. (f) Comparison of the PL lifetime decays in D_2_O and H_2_O.

The synthesized Au_18_(SG)_14_ nanoclusters (shown in Fig. 1a) exhibit a quantum yield of 7% in pure water with an emission peak at 660 nm and a broad excitation spectrum in the blue-green region (as depicted in Fig. 1b). Our primary objective is to gain a deeper understanding of their photophysical properties. To this end, we implement FCS to analyze the luminescence from a few nanoclusters in a well-defined confocal detection volume (Fig. 1c).^19^ The FCS analysis allows the quantification of various processes affecting the photoluminescence signal, as schematically depicted in Fig. 1d. The amplitude of the FCS correlation function is proportional to the inverse number of AuNCs present in the confocal detection volume, while the characteristic shape of the correlation function at different timescales provides information about different processes such as photon antibunching, blinking and Brownian diffusion as we will quantify later below.

A characteristic feature of AuNCs is the presence of the antibunching dip occurring at microsecond timescales in the FCS correlation function (Fig. 1d). For conventional organic fluorescent dyes with fluorescence lifetimes in the nanosecond range, this antibunching dip in the FCS function is rarely seen.^24,25^ Instead, Au_18_(SG)_14_ NCs have a luminescence lifetime in the microsecond range, so that the antibunching dip can be clearly resolved in the FCS correlation. The physical origin of this dip relates to the quantum nature of the single photon emitted by a single AuNC.^24,26–28^ The single photons are either transmitted or reflected at the 50/50 beamsplitter separating avalanche photodiodes, but the two photodiodes cannot detect a correlated signal simultaneously at a lag time shorter than the typical PL lifetime. The presence of the antibunching dip in the Au_18_(SG)_14_ FCS data highlights the quantum nature of the PL emission and the fact that photons are emitted one by one for each nanocluster. Said differently, there is only one emitting center per nanocluster; the Au_18_(SG)_14_ investigated here does not carry multiple emitting centers per AuNC. While the antibunching time includes a dependence on the excitation rate (see eqn (7) in the Materials and methods section), the observation of the antibunching dip is not expected to depend on the choice of the excitation wavelength, as is the luminescence lifetime.

To improve the luminescence brightness of AuNCs, we replace water H_2_O with heavy water D_2_O as the solvent. The rationale behind this choice is motivated by the fact that O–D stretching has a lower vibrational frequency compared to O–H stretching, thereby reducing solvent-mediated non-radiative decays when using D_2_O.^29,30^ The outcomes presented in Fig. 1e demonstrate a substantial 30% increase in PL intensity when D_2_O is used compared to H_2_O. This luminescence intensity gain is accompanied by a PL lifetime increase as shown in Fig. 1f. In H_2_O, the intensity-averaged lifetime is approximately 1190 ns, which increases to 1350 ns when D_2_O is used as buffer. This lifetime increase is reminiscent of the fluorescence lifetime gain observed with visible and near-infrared fluorescent dyes and is consistent with a reduction of the nonradiative decay rate constant due to the reduced quenching losses with D_2_O instead of H_2_O.^29,30^ In the following, all the experiments use D_2_O instead of H_2_O to maximize the PL intensity.

### FCS data of Au_18_(SG)_14_

**Fig. 2:**
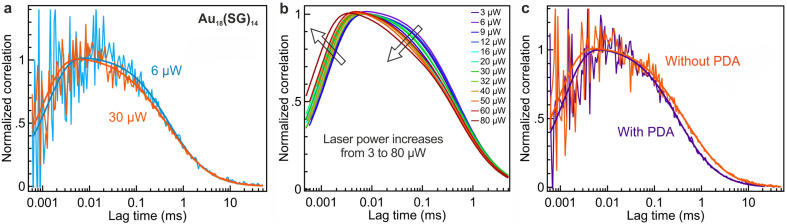
(a) Normalized FCS correlation and numerical fits of Au_18_(SG)_14_ in D_2_O at two different powers (6 μW and 30 μW). (b) Comparison of the numerical fits of the normalized FCS correlation of Au_18_(SG)_14_ in D_2_O with increasing the laser power from 3 μW to 80 μW. The trend in the numerical fits with increasing laser power is denoted by the arrow. (c) Comparison of the normalized FCS correlations and fits of Au_18_(SG)_14_ in the presence and absence of PDA at 20 μW. The measurements of Au_18_(SG)_14_ without PDA are performed in 98% v/v of D_2_O and the measurements of Au_18_(SG)_14_ with PDA are performed in a PBS buffer of pH 11. The excitation laser used is 557 nm.

Fig. 2 presents FCS data for the different samples tested on the confocal setup. We aim to probe the evolution of the FCS data with the laser power (Fig. 2a and b) as well as the influence of the outer ligand surrounding the AuNC (Fig. 2c). It has been reported in ref. 31 that the addition of pyridinedicarboxaldehyde (PDA) to glutathione-stabilized nanoclusters enhances the PL signal by forming a robust covalent imine bond between PDA and SG at pH 11. We have decided to apply a similar procedure to functionalize our Au_18_(SG)_14_ nanoclusters with PDA and compare their photophysical properties in the presence or absence of PDA using FCS.

Representative examples of correlation functions for Au_18_(SG)_14_ are shown in Fig. 2a at two different excitation powers and in Fig. 2c in the presence or absence of the ligand exchange procedure involving PDA. By fitting the correlation functions using eqn (1) (see Materials & methods), we gain insights into the diffusion kinetics and photophysical characteristics of Au_18_(SG)_14_. The FCS data allow the hydrodynamic radius *r*_H_ of Au_18_(SG)_14_ to be determined. For this, we use a separate calibration of the FCS diffusion time with Alexa Fluor 647 which is known to have a 0.7 nm hydrodynamic radius.^32^ By using the equation *r*_H_/*r*_Alexa647_ = *τ*_D_/*τ*_D(Alexa647)_, we obtain a hydrodynamic radius of 2.8 ± 0.5 nm for Au_18_(SG)_14_.

**Fig. 3:**
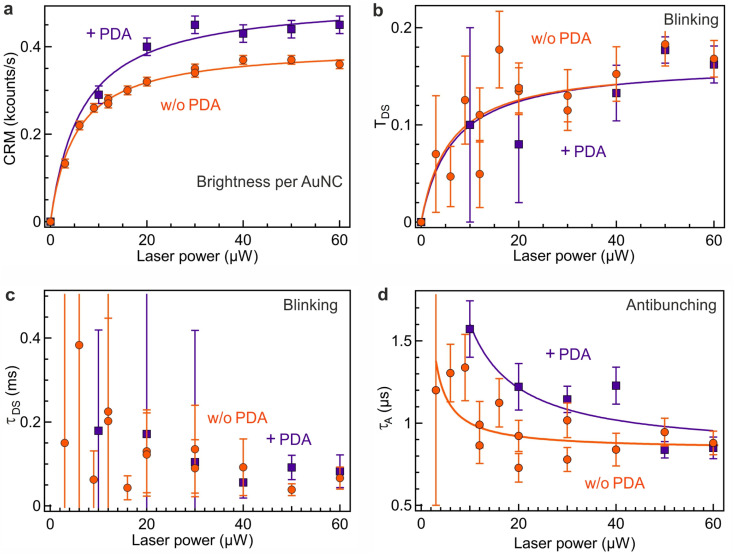
Comparison of different photophysical parameters of Au_18_(SG)_14_ obtained from the FCS data with increasing excitation laser power and in the presence or absence of PDA. (a) Fluorescence brightness per Au_18_(SG)_14_ with and without PDA. (b) Dark state amplitude *T*_DS_. (c) Dark state blinking time *τ*_DS_. (d) Antibunching time *τ*_A_.

Fig. 2b highlights a discernible change in the shape of the fitted curve at different power levels. This is attributed to the dependence of photophysical parameters with the laser power and the occurrence of PL saturation due to the finite PL lifetime. As the laser power is increased (Fig. 2b) the antibunching dip seen at submicrosecond timescales becomes narrower as a consequence of the reduction of the antibunching time *τ*_A_ when the laser power is increased (see eqn (7)). Similarly, the blinking time *τ*_DS_ gets shorter when the laser power is increased (as expected from eqn (6)) and the fraction of molecules in the dark state *T*_DS_ increases (see eqn (5)). The different fit results are summarized in Fig. 3 and S3.†

### Photophysical parameters of Au_18_(SG)_14_

Fig. 3 summarizes our main results assessing the photophysical characteristics of Au_18_(SG)_14_ nanoclusters and their evolution as a function of the excitation power and the presence of PDA. The luminescence brightness per nanocluster CRM follows the power dependence described by eqn (4) with a linear dependence as the laser power remains below the saturation power *P*_sat_ of 5.5 μW, and a saturation when the laser power exceeds *P*_sat_ (Fig. 3a). The presence of PDA induces a significant increase in the CRM of Au_18_(SG)_14_ nanoclusters. The saturation power in the presence of PDA is also slightly raised up to 6.5 μW.

The observed blinking properties of Au_18_(SG)_14_ nanoclusters have a moderate amplitude *T*_DS_ ranging from 0.04 to 0.2, contingent upon the power level (Fig. 3b), while the blinking time *τ*_DS_ remains in the order of 100 to 200 μs (Fig. 3c). The limited PL brightness per nanocluster (Fig. 3a) leads to a relatively large noise in the determination of the blinking parameters. Fig. S2† compares the fitting of the FCS data with and without taking into account the dark state blinking. The lower fitting residuals with the blinking model substantiate the occurrence of moderate dark state blinking in the Au_18_(SG)_14_ sample. The presence of PDA does not appear to modify the blinking properties. These properties come in stark contrast with the strong blinking properties observed among various DNA-encapsulated silver nanoclusters.^23^ While these silver nanoclusters were reported to undergo photoinduced charge transfer between the silver core and the encapsulating DNA molecules,^23^ our AuNCs remain largely free of photoinduced blinking.

In the microsecond range and below, all the FCS correlation functions exhibit a characteristic antibunching dip, with the respective antibunching time *τ*_A_ decreasing from from 1.5 μs to 0.8 μs as the laser power is increased (Fig. 3d). This evolution is expected from the model eqn (7). The occurrence of antibunching confirms that the detected photons are coming from a single quantum emitter and not a collective emission of multiple emitting centers. The presence of PDA increases the antibunching time, indicating a similar increase in the PL lifetime confirmed by TCSPC measurements and the higher saturation power seen for PDA in Fig. 3a. These findings are in correlation with previous research on gold nanoclusters functionalized with PDA.^31^ We can relate this PL lifetime increase to a reduction of the nonradiative decay rate constant in the presence of PDA which further protects and stabilizes the AuNC.

### PL enhancement using zero-mode waveguide nanoapertures

**Fig. 4:**
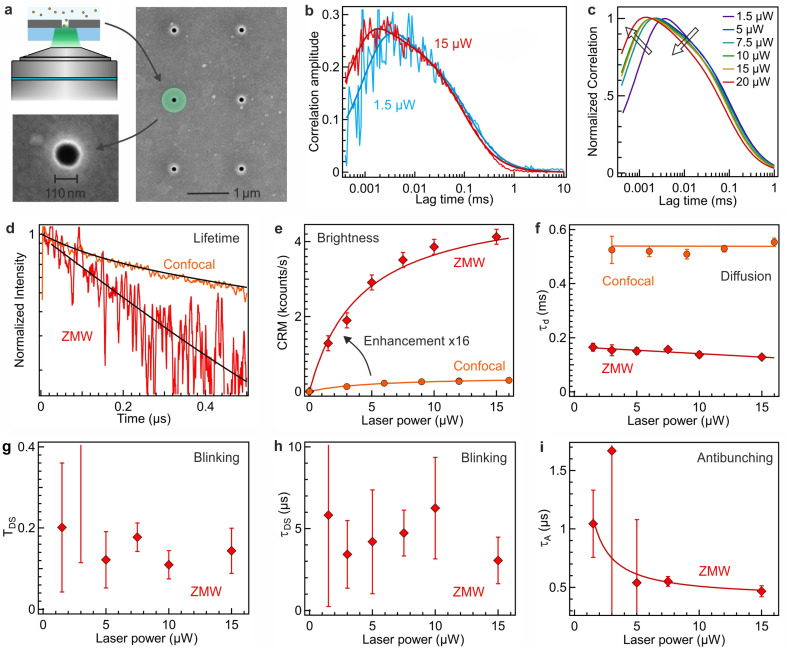
Enhancement of AuNC PL using zero-mode waveguide nanoapertures. (a) Scanning electron microscope (SEM) image of an aluminium ZMW aperture of 110 nm dimeter and its schematic representation at the microscope focus. (b) FCS correlation and numerical fits of Au_18_(SG)_14_ in ZMW at two different powers (1.5 μW and 15 μW) of 557 nm excitation. (c) Comparison of the numerical fits of the FCS correlation of Au_18_(SG)_14_ in a ZMW with increasing the laser power from 1.5 μW to 20 μW. The trends with increasing laser powers are denoted by the arrows. (d) PL lifetime decay traces of Au_18_(SG)_14_ in confocal (diffraction-limited) and ZMW nanoapertures. (e) Comparison of Au_18_(SG)_14_ brightness per nanocluster in the confocal and ZMW setup. (f) Comparison of diffusion times of Au_18_(SG)_14_ in the confocal and in the ZMW. (g–i) Evolution of different photophysical parameters of Au_18_(SG)_14_ in the ZMW obtained from the FCS data with increasing excitation laser power: (g) dark state amplitude *T*_DS_, (h) dark state blinking time *τ*_DS_ and (i) antibunching time *τ*_A_.

To further improve the PL brightness of AuNCs and demonstrate that nanophotonics can be used to enhance their PL properties,^33,34^ we use zero-mode waveguide (ZMW) nanoapertures. ZMWs are 110 nm diameter nanoapertures milled in an opaque aluminum film (Fig. 4a).^35–37^ As the ZMW diameter is well below the optical wavelength, light cannot propagate through the aperture and thus the light intensity is confined at the bottom of the ZMW.^38^ When a single ZMW is positioned at the microscope focus, it provides an effective way to confine light and locally overcome the diffraction limit. ZMWs have been reported to provide attoliter detection volumes together with 5 to 20× enhancement for the brightness of organic fluorescent dyes.^39,40^ Here, we demonstrate that the remarkable optical properties of ZMWs are fully applicable to AuNCs and can be used to further improve their brightness.

The correlation functions at different excitations powers are shown in Fig. 4b and c, where we retrieve the characteristic photodynamic features seen in the confocal configuration (Fig. 1d and 2). In the ZMW, the PL lifetime is reduced as a consequence of the modification of the electromagnetic environment of the emitter^39,40^ and becomes 1 μs (Fig. 4d). The PL brightness per AuNC is significantly enhanced with the ZMW as compared to the conventional confocal FCS method (Fig. 4e). At lower powers below saturation, the enhancement factor approaches 16×. As for organic fluorescent dyes, the brightness enhancement for AuNCs can be explained by a combination of local excitation intensity gain, quantum yield improvement and collection efficiency gain.^36,39,41^ The saturation power is ∼4 μW, lower than that of confocal (∼5.5 μW). While the confocal brightness remains around 400 counts per s at best (Fig. 3a), in the ZMW, PL brightness of 4000 counts per s is readily achieved, demonstrating the superior emission performance inside ZMWs.

The diffusion time *τ*_D_ in the ZMW amounts to 140 μs (Fig. 4f) and is lower than that of confocal measurements due to the smaller observation volume within the ZMW. As observed previously with fluorescent dyes, the reduction in the diffusion time *τ*_D_ is not proportional to the ratio of the illuminated areas, as the 3D shape of the ZMW nanoaperture requires additional time for the molecule to diffuse across the bottom of the ZMW.^39,40^

The blinking parameters *T*_D_ and *τ*_DS_ in the ZMW are shown in Fig. 4g and h. They display a moderate evolution with the laser power, which we relate to the occurrence of saturation at reduced powers and the larger statistical noise in the determination of the blinking dynamics. As for the confocal reference (Fig. 3b), we find that the blinking amplitude remains generally below 0.2, yet the blinking time appears to be accelerated to about 5 μs timescale. This apparent acceleration in the blinking dynamics can be related to the higher local excitation intensity inside the ZMW and to the presence of the metallic walls of the ZMW which promote nonradiative energy transfer to the metal. Lastly, we retrieve the characteristic antibunching dip in the FCS data, with the antibunching time summarized in Fig. 4i. In agreement with the PL lifetime reduction in the ZMW, the antibunching time is also reduced in a similar manner. Altogether, the data in Fig. 4 demonstrate that ZMWs can be used as a high efficiency platform for different applications of AuNCs.

## Conclusions

While AuNCs have attracted much attention as novel luminescent probes for biosensing and bioimaging, their photodynamic properties remained elusive as most studies focused on ensemble-averaged techniques. Here, we successfully implement fluorescence correlation spectroscopy to reveal the photoluminescence dynamic properties of Au_18_(SG)_14_ down to the submicrosecond timescale. Thanks to FCS, the PL brightness per nanocluster can be measured, enabling the determination of the influence of photoexcitation saturation as well as the role of ligands surrounding the nanocluster. We have exemplified that the presence of PDA can significantly promote the PL brightness per single emitter and increase its PL lifetime. FCS simultaneously allows the blinking dynamics to be recorded. Here we show that contrary to DNA-encapsulated silver nanoclusters,^23^ gold nanoclusters show minimal blinking, with amplitudes below 0.2 and characteristic times around 200 μs under the confocal conditions. This behavior enhances the potential applications of AuNCs in bioimaging. In addition, our FCS data show a clear antibunching peak at microsecond lag times. The observation of the antibunching phenomenon highlights the quantum nature of the PL process in the AuNC and importantly demonstrates that each AuNC behaves as a single quantum source. This means that each AuNC carries a single emitting center, or very much like quantum dots, the whole AuNC participates in the PL emission process. Finally, we demonstrate that ZMW nanoapertures can be used to further enhance by 16-fold the PL brightness of individual AuNCs. This proof-of-principle demonstration importantly unlocks the use of nanophotonics to tailor the emission properties of AuNCs. Gaining extra insights on the AuNC photodynamics beyond those accessible to ensemble-averaging techniques is an important step for the future development of AuNCs in biosensing and imaging applications, especially in the single-particle regime.

## Materials and methods

### Nanocluster synthesis protocol

Gold nanoclusters were synthesized using the protocol described in ref. 34; however the scale of synthesis was downscaled. Briefly, 100 mg of l-glutathione (GSH), 0.4 ml of methanol and 0.4 ml of water were mixed in a 25 ml round flask. After few minutes of dissolving GSH, HAuCl_4_·3H_2_O was added (200 μl, 0.6362 M) and mixed for another 10 min. During this time, a gradual discoloration can be observed – from an intense yellow to almost colorless. Then, the solution was diluted with methanol to 10 ml. Next, slow reduction was performed using methanolic solution of NaBH_3_CN (1.5 ml, 220 mM). The solution was left for mixing for 1 hour. A gradual color change from yellow to orange to dark brown was observed. After 1 hour the precipitate was collected and washed with MeOH three times through centrifugal precipitation (10 min/12 000 rpm). The collected sediment was dissolved in a small amount of water and washed three times with methanol/ethanol, each time centrifuged (10 min/12 000 rpm) to remove the remaining reaction precursors.

### Sample preparation

The gold nanocluster sample (Au_18_SG_14_) is diluted in deuterium oxide (D_2_O). D_2_O is purchased from Sigma-Aldrich and used as received. The v/v ratio of nanoclusters with D_2_O is 98% for confocal and 50% for ZMW measurements. As for ZMWs we are limited by the minimal μM concentration required to perform FCS experiments and cannot dilute the stock AuNC solution more than twice. Before use, the ZMW nanoapertures are cleaned with Milli-Q water followed by rinsing with 97% ethanol. Then the ZMW sample is exposed to UV for 5 min to remove any organic impurities. 2,6-Pyridine carboxaldehyde is purchased from Sigma-Aldrich. Synthesis of PDA-coated AuNCs is performed following a reported protocol.^31^ The confocal experiments with this PDA-coated sample are performed in PBS buffer at pH 11 as we found that this system is unstable in D_2_O at pH 7.

### FCS setup

All the fluorescence measurements are performed on a home-built confocal microscope setup. Au_18_NC samples are excited at 557 nm using an iChrome-TVIS laser (Toptica GmbH, pulse duration ∼ 3 ps). The repetition rate of the laser is 40 MHz. A multiband dichroic mirror (ZT 405/488/561/640rpc, Chroma) reflects the laser towards the microscope, and a Zeiss C-Apochromat 63×, 1.2 NA water immersion objective lens is used to focus the excitation light. The same objective lens collects the PL signal in an epifluorescence configuration. The PL beam then passes through the same multiband dichroic mirror. To block the laser back reflection an emission filter (ZET405/488/565/640mv2, Chroma) is used. The fluorescence signal is focused onto an 80 μm pinhole. Two avalanche photodiode APDs (PerkinElmer SPCM-AQR-13) separated by a 50/50 beam-splitter in a Hanbury-Brown–Twiss configuration record the emitted photons in the 650–750 nm spectral range. The photodiode outputs are connected to a time-correlated single photon counting (TCSPC) module (HydraHarp 400, Picoquant). The integration time for each FCS experiment was set to 40 minutes for the confocal experiments and 5 minutes for the ZMWs.

### FCS analysis

FCS traces are obtained from the cross-correlation of the fluorescence intensity time trace from two APDs. We time-gated and discarded photons within the 0–2 ns range out of the 25 ns pulse interval to reduce the background due to laser-induced backscattering and Rayleigh scattering (Fig. S1†). We used the following three-dimensional Brownian diffusion model with additional terms for blinking and anti-bunching effects to fit the FCS curves:^19^1
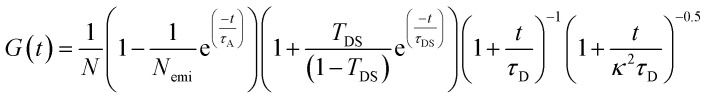
where *G*(*t*) is the cross-correlation function at time *t*, *N* is the total number of AuNCs in the observation volume, *N*_emi_ is the number of emitting species per AuNC, *τ*_D_ is the mean diffusion time, *T*_DS_ is the fraction of emitters in the dark state, *τ*_DS_ is the dark state blinking time, *τ*_A_ is the antibunching time and *κ* corresponds to the aspect ratio of the axial to the transversal dimension of the detection volume. Our different FCS data converge toward similar values *N*_emi_ around one, so for consistency in the analysis, we decided to keep the *N*_emi_ parameter at 1 for all the analysis. We have taken *κ* as 5 for confocal and *κ* as 1 for ZMW nanoapertures based on our past results as this fits well with our experimental FCS data. To extract different photophysical parameters from eqn (1) we fit the experimental data from 0.01 μs to 100 ms. From the fitted FCS curve we extract the value of *N*, which is then corrected for the background (*B*) using eqn (2) to determine the background corrected value for *N*_corr_2
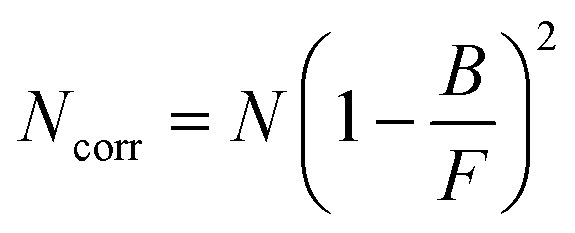
Here, *B* is the measured background and *F* is the total PL intensity. *N*_corr_ is defined as the mean number of detected fluorescent molecules in the observation volume averaged over the duration of the experiment. We also calculate the brightness per nanocluster CRM using3
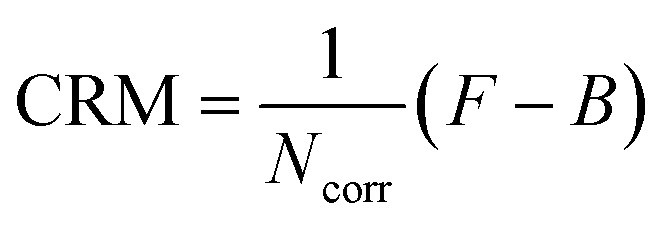


The evolution of the brightness per nanocluster CRM is given by^18^4
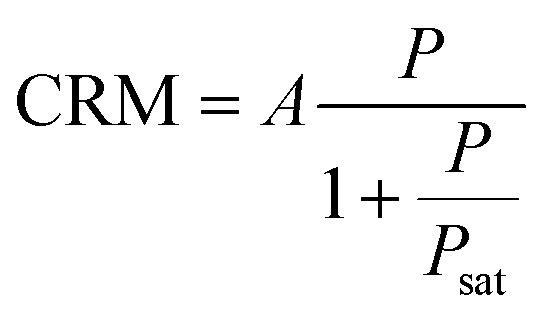
Here, *A* = *ηϕσρ* is the product of collection efficiency *η*, the quantum yield *ϕ*, the excitation cross-section *σ* and a constant proportionality parameter *ρ* to accommodate for the different units while expressing the excitation power *P* in microwatts. *P*_sat_ is the saturation power of the AuNC.

The dark state amplitude *T*_DS_ follows a similar power dependence given by^42–44^5
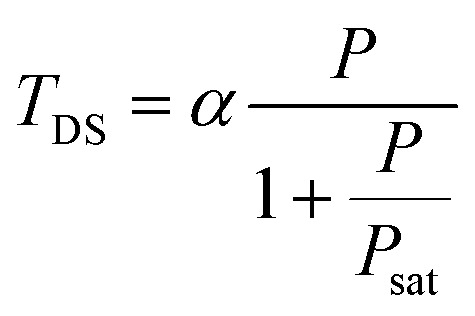
where 
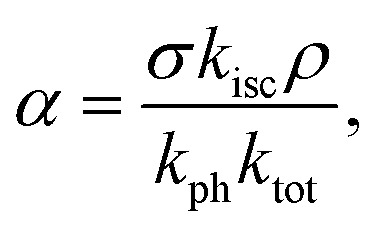
*k*_isc_ and *k*_ph_ are the rate constants of inter-system crossing and dark state de-excitation respectively and *k*_tot_ is the total de-excitation rate constant.

The blinking time *τ*_DS_ is related to the different decay rate constants as^42–44^6
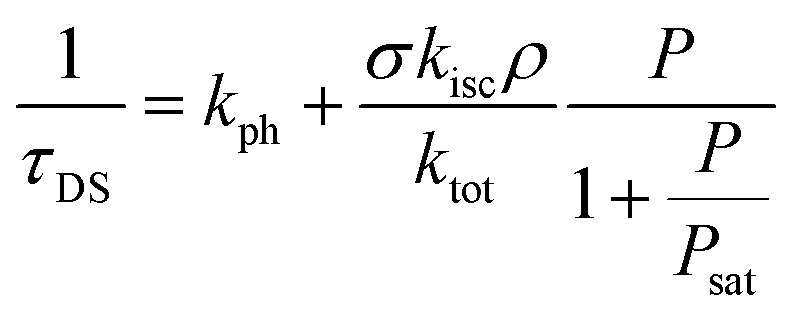


Lastly, the antibunching time *τ*_A_ is given by^26^

7 1/*τ*_A_ = *k*_tot_ + *σρP*

### Zero mode waveguide fabrication

The focused ion beam (FIB) technique is used to fabricate zero-mode waveguide nanoapertures into an aluminum film of thickness 100 nm deposited on a glass coverslip. The deposition of aluminum on a glass coverslip is carried out by electron beam evaporation (Bühler Syrus Pro 710). The chamber pressure is set at 5 × 10^−7^ mbar and the aluminum is deposited at a rate of 10 nm s^−1^ to achieve the optimum performance of our ZMW. A focused gallium beam (10 pA current and 30 kV voltage) with a resolution of 10 nm is then used to mill ZMW nanoapertures of diameter 110 nm into the aluminum film. A 12 nm-thick silica layer is deposited with plasma-enhanced chemical vapour protection (Oxford Instruments PlasmaPro NGP80) to protect the aluminum sample.

## Data availability

The data that support the findings of this study data are available from the corresponding author upon request.

## Conflicts of interest

The authors declare no conflict of interest.

## Supplementary Material

NA-006-D3NA00869J-s001
