# Supplementary material for: Unveiling the photoluminescence dynamics of gold nanoclusters with fluorescence correlation spectroscopy
Source: Nanoscale Adv. 2023 Dec 4;6(2):570–7. doi: 10.1039/d3na00869j (PMC10790814; doi:10.1039/d3na00869j)
Supplement: NA-006-D3NA00869J-s001 [file NA-006-D3NA00869J-s001.pdf]

Electronic Supplementary Material (ESI) for Nanoscale Advances.
This journal is © The Royal Society of Chemistry 2023

Supporting Information for

Unveiling the photoluminescence dynamics of gold nanoclusters with fluorescence correlation spectroscopy

Malavika Kayyil Veedu,[1] Julia Osmólska,[2] Agata Hajda,[2] Joanna Olesiak-Bańska,[2] Jérôme Wenger[1,*]

[1] *Aix Marseille Univ, CNRS, Centrale Marseille, Institut Fresnel, AMUTech, 13013 Marseille, France*

[2] *Institute of Advanced Materials, Wroclaw University of Science and Technology, Wrocław, Poland*

* *Corresponding author: jerome.wenger@fresnel.fr*

Contents:

S1. **TCSPC decays of background**

S2. **Comparison of FCS fitting with and without the dark state blinking term**

S3. **Supplementary FCS fit results**

S1. TCSPC decays of background

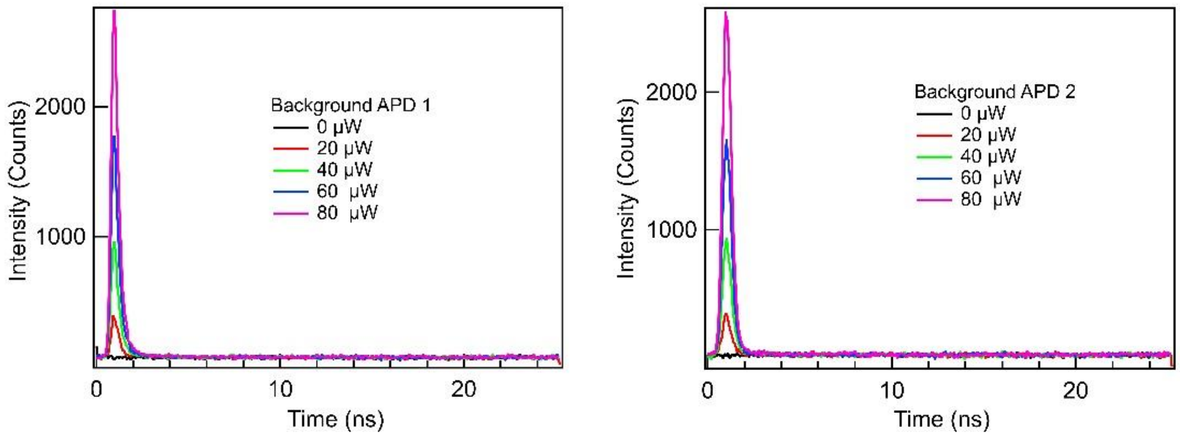

Figure S1. TCSPC measurement of D_2O background at different powers. Here the fluorescence intensity counts in the first 2 ns are mainly due to laser-induced backscattering and Raman scattering. Hence we time gate intensity counts at the first 2 ns to remove the scattering peak and reduce the background.

S2. Comparison of FCS fitting with and without the dark state blinking term

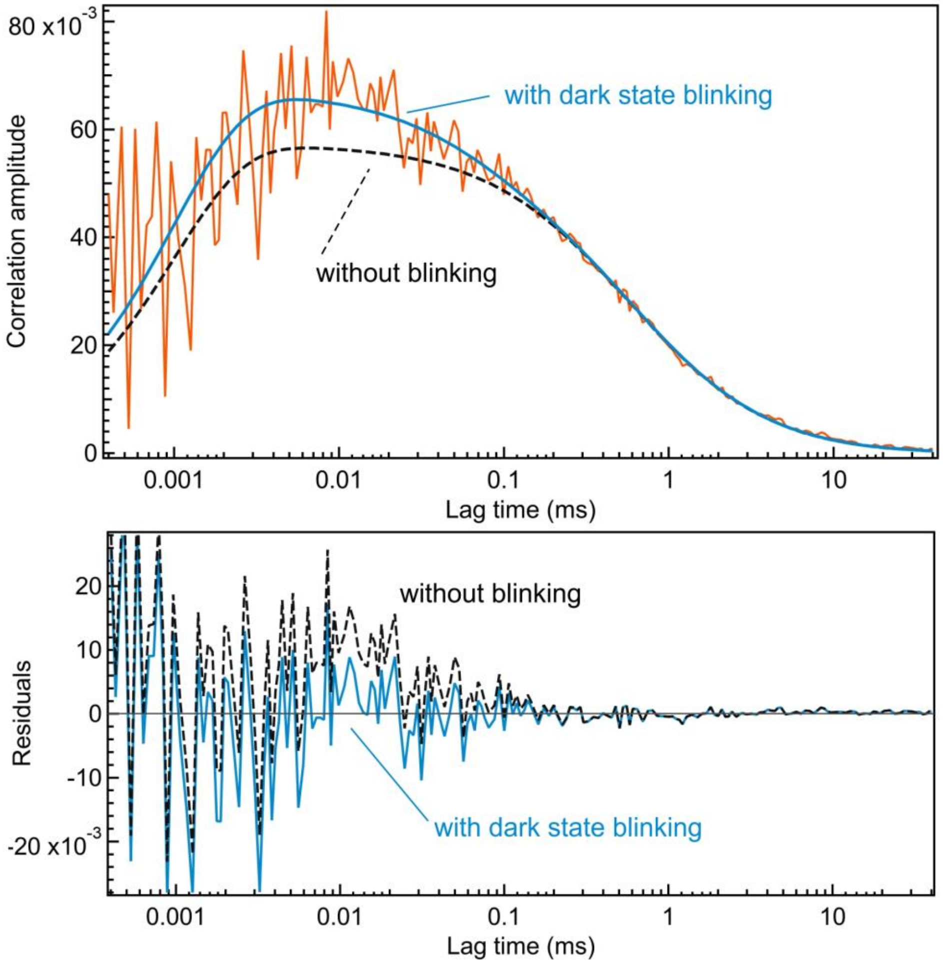

Figure S2. Comparison of FCS numerical fits with (solid blue line) and without (dashed gray line) including the term $\left(1+\frac{T_{DS}}{(1-T_{DS})}e^{\left(\frac{-t}{\tau_{DS}}\right)}\right)$ accounting for the dark state blinking. The lower graph shows the fit residuals. The excitation power is 30 μW.

S3. Supplementary FCS fit results

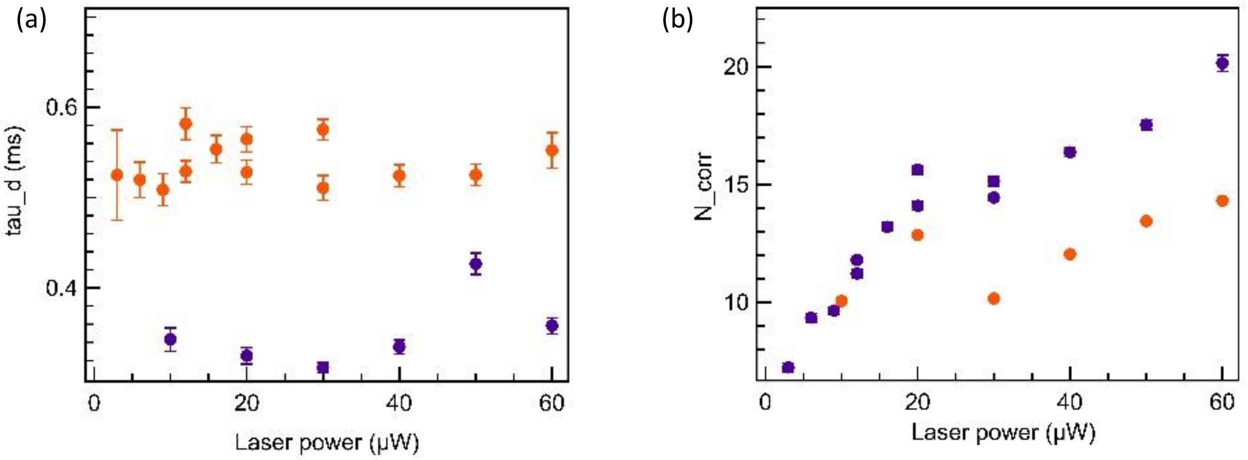

Figure S3. a) Diffusion time of $Au_{18}(SG)_{14}$ with/without PDA at different powers. b) Variation in the number of AuNC in the confocal detection volume $N_{\_corr}$ with/without PDA with power.
